# Linc‐ROR promotes the progression of breast cancer and decreases the sensitivity to rapamycin through miR‐194‐3p targeting MECP2

**DOI:** 10.1002/1878-0261.12700

**Published:** 2020-05-24

**Authors:** Qian Zhou, Juan Guo, Wenjie Huang, Xiaosi Yu, Chen Xu, Xinghua Long

**Affiliations:** ^1^ Department of Laboratory Medicine Zhongnan Hospital of Wuhan University China

**Keywords:** breast cancer, ceRNA, linc‐ROR, next‐generation sequencing, rapamycin

## Abstract

linc‐ROR is reported to be a potential biomarker of breast cancer, but the detailed mechanism of linc‐ROR‐mediated breast cancer regulation has not been fully studied. We aimed to explore how linc‐ROR affects proliferation, metastasis, and drug sensitivity in breast cancer. Cell lines in which linc‐ROR was overexpressed or knocked down were constructed, and the cell proliferation, colony formation, cell migration, and invasion abilities of these lines were explored. A CCK‐8 assay was performed to determine the sensitivity of the breast cancer cells to rapamycin. Next‐generation sequencing was conducted to explore the detailed regulatory mechanism of linc‐ROR; differentially expressed RNAs in the linc‐ROR‐overexpressing cell line compared with the negative control were screened out, and their target genes were chosen to perform Gene Ontology analysis, Kyoto Encyclopedia of Genes and Genomes analysis, protein–protein interaction network analysis, and competing endogenous RNA (ceRNA) network analysis. The ceRNA mechanism of linc‐ROR for miR‐194‐3p, which targets MECP2, was determined through dual‐luciferase reporter assay, RT–qPCR, western blot, and rescue experiments. Finally, we found that linc‐ROR was upregulated in breast tumor tissues. linc‐ROR promoted the cell proliferation, colony formation, cell migration, and invasion of breast cancer and decreased the sensitivity of breast cancer cells to rapamycin. The overexpression of linc‐ROR triggered changes in the whole transcriptome of breast cancer cells, and a total of 85 lncRNAs, 414 microRNAs, 490 mRNAs, and 92 circRNAs were differentially expressed in the linc‐ROR‐overexpressing cell line compared with the negative control. Through a series of bioinformatic analyses, the ‘linc‐ROR/miR‐194‐3p/MECP2’ ceRNA regulatory axis was confirmed to be involved in the linc‐ROR‐mediated progression and drug sensitivity of breast cancer. In conclusion, linc‐ROR serves as an onco‐lncRNA in breast cancer and promotes the survival of breast cancer cells during rapamycin treatment by functioning as a ceRNA sponge for miR‐194‐3p, which targets MECP2.

## Graphical abstract

We found that linc‐ROR functioned as an onco‐lncRNA in breast cancer. Through the whole transcriptome sequencing and bioinformatic analyses, the ‘linc‐ROR/miR‐194‐3p/MECP2’ axis was screened out. Finally, it was confirmed that linc‐ROR promoted the progression of breast cancer and decreased the sensitivity of breast cancer cells to rapamycin by functioning as a ceRNA sponge for miR‐194‐3p, which targets MECP2.

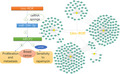

**Abbreviations**
ceRNA: competing endogenous RNA
DE‐RNAs: differentially expressed RNAs
EMT: epithelial‐to‐mesenchymal transition
ER: estrogen receptor
FPKM: fragments per kilobase of transcript sequence per millions
GO: Gene Ontology
KEGG: Kyoto Encyclopedia of Genes and Genomes
linc‐ROR: long intergenic non‐protein‐coding RNA, regulator of reprogramming
lncRNA: long noncoding RNA
MECP2: methyl‐CpG‐binding protein 2
miRNA: microRNA
PPI: protein–protein interaction
RT–qPCR: real‐time quantitative polymerase chain reaction
SEM: standard error of the mean

## 1 Introduction

Breast cancer is the most common invasive cancer in females and the second leading cause of female cancer‐related death (Siegel *et al*., 2019). Tumor metastasis, recurrence, and drug resistance are important factors that hinder effective treatment. Compared with traditional treatments, molecular targeting therapy seems to hold more promise for the prevention and cure of breast cancer.

Noncoding RNAs are increasingly proven to play important roles in breast cancer. Long noncoding RNAs (lncRNAs) are a class of noncoding RNA molecules with a length of 200–100 000 nucleotides (Guttman *et al*., 2009) that can regulate gene expression by chromosome remodeling, transcriptional regulation, and post‐transcriptional processes (Shi *et al*., 2013). lncRNAs can be derived from protein‐coding genes, pseudogenes, and DNA sequences between protein‐coding genes. linc‐ROR (long intergenic non‐protein‐coding RNA, regulator of reprogramming) is a kind of intergenic noncoding RNA that has been shown to be dysregulated in many types of cancers, including breast cancer (Pan *et al*., 2016). Overexpressed linc‐ROR was reported to be a potential biomarker for the diagnosis and dynamic monitoring of breast cancer, and the expression levels of linc‐ROR are significantly higher in breast cancer tissues and plasma than in controls (Zhao *et al*., 2017). linc‐ROR is important in regulating epithelial‐to‐mesenchymal transition (EMT) and can promote breast cancer progression and metastasis through the regulation of miRNAs (Hou *et al*., 2014). linc‐ROR also functions as an onco‐lncRNA to promote the estrogen‐independent growth of ER‐positive breast cancer and to contribute to tamoxifen resistance (Peng *et al*., 2017). linc‐ROR is an important marker for multidrug resistance in breast cancer, and its upregulation is important for chemotherapy tolerance (Chen *et al*., 2016a), and it confers the resistance of breast cancer cells to gemcitabine by silencing miR‐34a expression (Chen *et al*., 2016b).

MicroRNAs (miRNAs) are a class of small noncoding RNA molecules with 20–24 nucleotides that regulate gene expression at the post‐transcriptional level (Bushati and Cohen, 2007). miRNAs can downregulate the expression of target genes by forming a noncomplementary hybrid double chain with the 3′‐UTR sequence of the target mRNA; then, miRISC (miRNA‐induced silencing complex) tightly binds to the hybrid double chain to specifically inhibit gene expression (Carthew and Sontheimer, 2009). Circular RNAs (circRNAs) are a class of enclosed cyclic noncoding RNAs that are abundant in the binding sites of miRNAs. Both lncRNAs and circRNAs can act as miRNA sponges and eliminate the inhibitory effect of miRNAs on their target genes by binding to miRNAs, thus increasing the expression of target genes and triggering a corresponding biological effect (Tay *et al*., 2014); this mechanism is called the competitive endogenous RNA (ceRNA) mechanism. linc‐ROR has been proven to act as a ceRNA for miR‐145 to suppress proliferation and invasion in prostate cancer stem cells (Liu *et al*., 2017), function in the differentiation of endometrial cancer stem cells (Zhou *et al*., 2014), and promote radioresistance in hepatocellular carcinoma cells (Chen *et al*., 2018). In addition, linc‐ROR can act as a ceRNA for both miR‐145 and miR‐138 to promote the osteogenic differentiation of mesenchymal stem cells (Feng *et al*., 2018). However, studies of the role of linc‐ROR in breast cancer are not yet sufficient, especially studies of the ceRNA mechanism of linc‐ROR. Therefore, we aimed to explore whether linc‐ROR could act as a sponge for microRNAs to affect the progression, metastasis, and drug sensitivity of breast cancer.

## 2 Materials and methods

### 2.1 Breast tissues and breast cancer cell lines

Thirty‐six samples of human breast tissues, including 20 tumor tissue samples and 16 paracarcinoma tissue samples, were collected from Hubei Cancer Hospital. The study methodologies of these samples conformed to the standards set by the Declaration of Helsinki and approved by the local ethics committee. Meanwhile, the experiments were undertaken with the understanding and written consent of each subject. Among these samples, 20 tumor tissue samples were collected from 20 females, ranging in age from 38 to 66 years (mean ± SD: 51.2 ± 7.0 years), and 16 paracarcinoma tissue samples were collected from 16 females, ranging in age from 36 to 68 years (mean ± SD: 50.6 ± 8.7 years).

The human breast cancer MCF‐7 cell line and the human embryonic kidney 293T cell line were purchased from American Type Culture Collection (ATCC, Manassas, VA, USA). Both of the two cell lines were stably cultured in Dulbecco's modified Eagle medium (DMEM; Hyclone, Logan, UT, USA) containing 10% FBS (Gibco, Grand Island, NY, USA) and 1% penicillin/streptomycin (Gibco) in an incubator with a constant temperature of 37 °C and 5% CO_2_.

### 2.2 RNA extraction, reverse transcription, and RT–qPCR

Total RNA was extracted from the tissues and cells by TRIzol reagent (Ambion, Austin, TX, USA), and the concentration, purity, and integrity of the extracted RNAs were determined by a NanoDrop 2000 spectrophotometer (Thermo Scientific, Waltham, MA, USA). Then, these RNAs were reverse‐transcribed to cDNA by the ReverTra Ace qPCR RT Kit (TOYOBO, Osaka, Japan) following the manufacturer's instructions. The obtained cDNAs were used as templates to be amplified by using the UItraSYBR Mixture (CWBIO, Jiangsu, China) on the Real‐Time PCR CFX96 Detection System (Bio‐Rad, Hercules, CA, USA) to perform RT–qPCR. U6 snRNA was used as the internal control for the normalization of the miRNA expression, and GAPDH was used for the normalization of the lncRNA and mRNA expression. The primers involved in the experiment are shown in Table 1.

**Table 1:** Primers used in the expression detection of RNAs by RT–qPCR. Primer mix: It contains random primer and oligo(dT) primer, applied by ReverTra Ace qPCR RT kit (TOYOBO).

	RT primer (5′–3′)	Forward primer (5′–3′)	Reverse primer (5′–3′)
GAPDH	Primer mix	GGTCTCCTCTGACTTCAACA	GTGAGGGTCTCTCTCTTCCT
linc‐ROR	Primer mix	CTCCAGCTATGCAGACCACTC	GTGACGCCTGACCTGTTGAC
MECP2	Primer mix	GCCGAGAGCTATGGACAGCA	CCAACCTCAGACAGGTTTCCAG
mTOR	Primer mix	CTGGGACTCAAATGTGTGCAGTTC	GAACAATAGGGTGAATGATCCGGG
U6	AACGCTTCACGAATTTGCGT	CTCGCTTCGGCAGCACA	AACGCTTCACGAATTTGCGT
miR‐194‐3p	GTCGTATCCAGTGCAGGGTCCGAGGTATTCGCACTGGATACGACCAGATA	GCCAGTGGGGCTGCTGT	AGTGCAGGGTCCGAGGTATT

### 2.3 Cell transfection

The stably cultured MCF‐7 cells were diluted to 3 × 10^4^ mL^−1^, evenly plated in two T25 culture vials (Corning, NY, USA), and cultured in 5 mL DMEM containing 10% FBS for 24 h. Then, lentiviral vectors packaged with linc‐ROR/negative control plasmids (Viraltherapy Technologies Co., Ltd., Wuhan, China), which expressed green fluorescent protein and conveyed puromycin resistance, were transfected into two groups of MCF‐7 cells. The ratio of viruses to cells was 70. After 24 h, the stably transfected cells were screened with 2 μg·mL^−1^ puromycin for one week. Then, the linc‐ROR‐overexpressing breast cancer cells (LV‐linc‐ROR) and a negative control (LV‐NC) were constructed. To verify the transfection efficiency, a fluorescence microscopy was used to determine the expression of green fluorescent protein in LV‐linc‐ROR and LV‐NC, and RT–qPCR was performed to detect the expression level of linc‐ROR in the two groups of cells. In addition, the linc‐ROR knockdown cell line (si‐linc‐ROR) and its negative control (si‐NC) were constructed by the transfecting Ribo™ lncRNA Smart Silencer (RiboBio, Guangzhou, China). RT–qPCR was used to determine the transfection efficiency.

### 2.4 Cell proliferation and drug sensitivity assay

The linc‐ROR overexpression (LV‐linc‐ROR, LV‐NC) and knockdown (si‐linc‐ROR, si‐NC) cells were plated in 96‐well plates (Corning) at a density of 3 × 10^3^ per well in 100 μL DMEM containing 10% FBS. After culturing for 0, 24, 48, and 72 h, 10 μL Cell Counting Kit‐8 (CCK‐8; Dojindo, Kumamoto, Japan) reagent was added to the corresponding wells and incubated for 3 h. Then, the absorbance was measured at a wavelength of 450 nm by a Multiskan FC microplate reader (Thermo Fisher, Waltham, MA, USA). The absorbance at each time point was normalized to that at 0 h, and all the data were presented in chronological order to show the cell proliferation status.

In addition, a drug sensitivity assay was conducted by using the CCK‐8 reagent. LV‐linc‐ROR and LV‐NC were seeded in 96‐well plates for 24 h, and then, rapamycin (Solarbio, Beijing, China) was added to the corresponding wells at concentrations of 0, 5, 10, 20, 50, and 100 μm. The absorbance at 450 nm was detected after 72 h.

### 2.5 Plate colony formation assay

One hundred individual LV‐linc‐ROR or LV‐NC cells were evenly plated in each Petri dish (60 mm; Corning) and cultured in DMEM containing 10% FBS for two weeks. Since the colonies formed in the linc‐ROR‐overexpressing group were generally larger than those in the linc‐ROR knockdown group, for clarity, 200 individual si‐linc‐ROR and si‐NC cells were plated in each Petri dish and cultured in DMEM containing 10% FBS for two weeks. The colonies were carefully bathed twice with PBS (Hyclone) and then fixed with 4% paraformaldehyde for 15 min. After that, the fixed cell colonies were stained with 0.1% crystal violet for 30 min.

### 2.6 Wound healing assay

A total of 5 × 10^5^ cells were plated in 6‐well plates and cultured in DMEM containing 3% FBS. After the cells adhered, a vertical scratch was made to simulate a wound by using a 10‐μL pipette tip. Then, photographs were taken in the same area of scratches every 12 h for 2 days under a microscope at 10× magnification and the wound healing rate at 0, 12, 24, 36, and 48 h was calculated as follows: wound healing rate = healing area/initial wound area.

### 2.7 Transwell migration and invasion assay

A 24‐well transwell chamber with 8.0‐μm‐pore membranes (Corning) was used to perform the transwell assay. First, the chamber was coated with (for the invasion assay) or without (for the migration assay) 10 μL of Matrigel (Corning), and incubated at 37 °C to solidify the Matrigel. Then, 10 μL of cells, cultured in serum‐free DMEM, was plated in the chamber at a density of 1.0 × 10^5^ cells/mL, and 600 μL DMEM containing 10% FBS was added to the 24‐well plates under the chamber. After 24 h, the cells above the membrane in the chamber were removed with cotton swabs, and then, cells that had already migrated or invaded to the bottom of the membrane were fixed with 4% paraformaldehyde for 15 min and stained with 0.1% crystal violet for 30 min. The pictures of theses stained cells were taken under a microscope at 10× magnification, and the migrated or invaded cells were counted to perform a quantitative analysis.

### 2.8 Next‐generation sequencing and bioinformatic analysis

#### 2.8.1 Next‐generation sequencing and the screening of differentially expressed RNAs

Total RNA was extracted from the LV‐linc‐ROR and LV‐NC using TRIzol reagent and sent to LC‐BIO (Hangzhou, China) to perform next‐generation sequencing. The quantity and purity of RNA were determined to have a RIN > 7.0 by a Bioanalyzer 2100 instrument (Agilent, Palo Alto, CA, USA). To conduct whole transcriptome sequencing, two sequencing libraries were constructed: small RNA libraries of less than 50 nt (microRNA) and RNA‐chain‐specific libraries that deplete ribosomal RNA and RNA greater than 200 nt (mRNA, lncRNA, circRNA). Illumina HiSeq 2500 (Illumina, San Diego, CA, USA) was used to perform single‐end sequencing (36 or 50 bp) for microRNAs, and Illumina HiSeq 4000 (Illumina) was used to perform paired‐end sequencing [an average of 300 bp (±bp)] for mRNAs, lncRNAs, and circRNAs.

The reads obtained from sequencing were put into Cutadapt (Martin, 2011) to remove the ones that contained adapter contamination, undetermined bases, and low‐quality bases. After verifying the sequence quality by using FastQC (http://www.bioinformatics.babraham.ac.uk/projects/fastqc/), the reads were mapped to *Homo sapiens* genome by using Bowtie2 (Langmead and Salzberg, 2012) and Tophat2 (Kim *et al*., 2013).

For mRNA and lncRNA, String Tie (Pertea *et al*., 2015) was used to assemble the mapped reads of each sample. Then, Perl scripts was used to merge all the transcriptomes from the *H. sapiens* samples to reconstruct a comprehensive transcriptome. The expression levels of all the transcripts, including mRNAs and lncRNAs, were determined by calculating the FPKM (Fragments per kilobase of transcript sequence per millions) using String Tie (Pertea *et al*., 2015). Then, the differentially expressed mRNAs and lncRNAs were screened out by the R package Ballgown (Frazee *et al*., 2015) according to the criteria of |log_2_ (fold change)| > 1 and *P* value < 0.05.

For circRNA, TopHat‐fusion (Kim and Salzberg, 2011) was used to map the remained reads to the genome. The mapped reads were *de novo* assembled to circRNAs by CIRCExplorer (Zhang *et al*., 2016), and then, back splicing reads were identified in the unmapped reads by TopHat‐fusion (Kim and Salzberg, 2011) and CIRCExplorer (Zhang *et al*., 2016). The circRNA expression levels from the different samples were calculated by scripts in house. And comparisons with a *P* < 0.05 were screened out as differentially expressed circRNAs by R package‐edgeR (Robinson *et al*., 2010).

For microRNA, ACGT101‐miR was used to remove the adapter dimers, junk, low complexity, common RNA families (rRNA, tRNA, snRNA, and snoRNA), and repeats. Then, miRbase 21.0 (Kozomara *et al*., 2018) and BlAST search were used to identify known microRNAs and novel 3p‐ and 5p‐derived microRNAs. The expression of microRNAs was analyzed according to normalized deep‐sequencing counts. Differentially expressed microRNAs were determined by *P* < 0.01 or *P* < 0.05 depending on the different statistical tests in the experimental design.

#### 2.8.2 GO and KEGG pathway analysis, ceRNA analysis, and PPI network analysis

The 100 most significantly differentially expressed lncRNAs, circRNAs, microRNAs, and mRNAs were depicted in a heatmap. Gene Ontology (GO) analysis and Kyoto Encyclopedia of Genes and Genomes (KEGG) pathway analysis of target genes were conducted using the R package. The ‘lncRNA‐microRNA‐mRNA’ and ‘circRNA‐microRNA‐mRNA’ ceRNA regulatory cascades were built by local Perl scripts. Then, DAVID (Huang *et al*., 2009) was used to perform the GO and KEGG analyses of the target genes involved in the ceRNA networks. The ceRNA network containing linc‐ROR was visualized by using Cytoscape software (Shannon *et al*., 2003) . Protein–protein interaction (PPI) network analysis was performed by using STRING (Szklarczyk *et al*., 2019). UALCAN (Chandrashekar *et al*., 2017) was used to analyze the effect of MECP2 on the survival curves of breast cancer patients and compare the MECP2 expression in breast cancer tissues with that in normal tissues.

### 2.9 Dual‐luciferase reporter assay

The complete sequence of linc‐ROR was amplified by using a high‐fidelity enzyme (MCLAB, San Francisco, CA, USA) to perform PCR, and the pmirGLO Dual‐luciferase miRNA Target Expression Vector (Promega, Madison, WI, USA) was digested by the Sac I (NEB, Ipswich, MA, USA) and XhoI (NEB) enzymes. Then, these two parts were ligated into a recombinant plasmid by the ClonExpress II One Step Cloning Kit (Vazyme, Nanjing, China). The recombinant linc‐ROR‐WT plasmid was verified by sequencing. The predicted binding sites between linc‐ROR and miR‐194‐3p were mutated by PCR (PrimeSTAR GXL DNA Polymerase; Takara, Kusatsu, Shiga, Japan) to construct the linc‐ROR‐MUT plasmid. Likewise, the 3′‐UTR of MECP2 was amplified by PCR (PrimeSTAR GXL DNA Polymerase; Takara), and then, MECP2‐WT and MECP2‐MUT were constructed as mentioned above. The primers used are shown in Table 2.

**Table 2:** Primers used in linc‐ROR‐WT/MUT and MECP2‐WT/MUT plasmid construction.

	Forward primer (5′–3′)	Reverse primer (5′–3′)
linc‐ROR‐WT	CTAGTTGTTTAAACGAGCTCGGTGAAATAAACAGCCATGT	CGACTCTAGACTCGAGGCTGTTTCTACATTTTATTTTTTGA
linc‐ROR‐MUT①	GCCCATCGCTAGAGTAAGTGAAACACAGGGACCACCTGCCTC	TTACTCTAGCGATGGGCCAGCGCAGGCTCTTTCTCTCCTGTGGTTTC
linc‐ROR‐MUT②	AATCTCTATTGGGAGCCAAATCGGACTGTCCAACTCACC	GGCTCCCAATAGAGATTTGAAATTGGTGAGATGTTTCTTGGGCTGGTTGG
MECP2‐WT	TCTAGTTGTTTAAACGAGCTCGTTCCTACCATGGAGTGGGTCTG	CAGGTCGACTCTAGACTCGAGGCCCAGGATAGAGGAGACAAAGC
MECP2‐MUT	TCCTGAAAGTCCATAGCCCCTCTCCCCGCAGT	GCTATGGACTTTCAGGAGCCAAACCTCTTGGC

Then, 4 μg of Linc‐ROR‐WT/MUT or MECP2‐WT/MUT and 100 nm miR‐194‐3p mimic or NC mimic (RiboBio) were cotransfected into HEK‐293T cells. After culturing for 48 h, the cells were fully lysed and collected to detect Firefly luciferase value and Renilla luciferase value by the Dual‐Luciferase Reporter Gene Assay Kit (Beyotime, Shanghai, China) under the Promega GloMax 20/20 Luminescence detector. The RLU ratio (firefly fluorescence value/Renilla fluorescence value) was calculated.

### 2.10 Western blot analysis

Total protein was obtained from the cell lines by using RIPA lysis buffer (Beyotime) containing 1 mm PMSF (Beyotime). The concentration of the extracted protein was determined by Enhanced BCA Protein Assay Kit (Beyotime). Twenty microgram protein from each cell line was electrophoresed in a 5% stacking gel at 60 V for 30 min, separated on a 6% (for the mTOR protein) or 10% (for the GAPDH and MECP2 proteins) SDS/PAGE gel at 110 V for 70 min, and then transferred to a PVDF membrane with a pore diameter of 0.22 μm (Biosharp, Hefei, China) at constant 250 A for 2 h. Then, the PVDF membranes containing the proteins were incubated with 5% BSA (BioFroxx, Thuringen, Germany) for 1.5 h, followed by incubation at 4 °C overnight with primary antibodies against mTOR (ABclonal, A2445, Wuhan, China), MECP2 (ABclonal, A5694), and GAPDH (ABclonal, AC033). TBST buffer was used to wash the membranes. Then, the membranes that contained GAPDH were incubated with HRP goat anti‐mouse IgG (H+L) (ABclonal, AS003), and the membranes that contained MECP2 and mTOR were incubated with HRP goat anti‐rabbit IgG (H+L) (ABclonal, AS014) for an hour at room temperature. After washing 10 min for four times with TBST buffer, the protein bands were visualized using an ECL Kit (Servicebio, Wuhan, China) and detected by Tanon 5200. Eight‐bit images were taken and analyzed by Image J (National Institutes of Health, Bethesda, MD, USA) to calculate the gray value.

### 2.11 Statistical analysis

GraphPad Prism 6 (GraphPad Software Inc., San Diego, CA, USA) was used to perform statistical analysis. The differences among groups were analyzed by the unpaired Student's *t*‐test, and the correlation between the expression of linc‐ROR and miR‐194‐3p was analyzed by linear regression. *P* < 0.05 was considered significant.

## 3 Results

### 3.1 linc‐ROR promotes the progression of breast cancer and decreases the sensitivity of breast cancer cells to rapamycin

#### 3.1.1 linc‐ROR is upregulated in breast tumor tissues

**Fig. 1:**
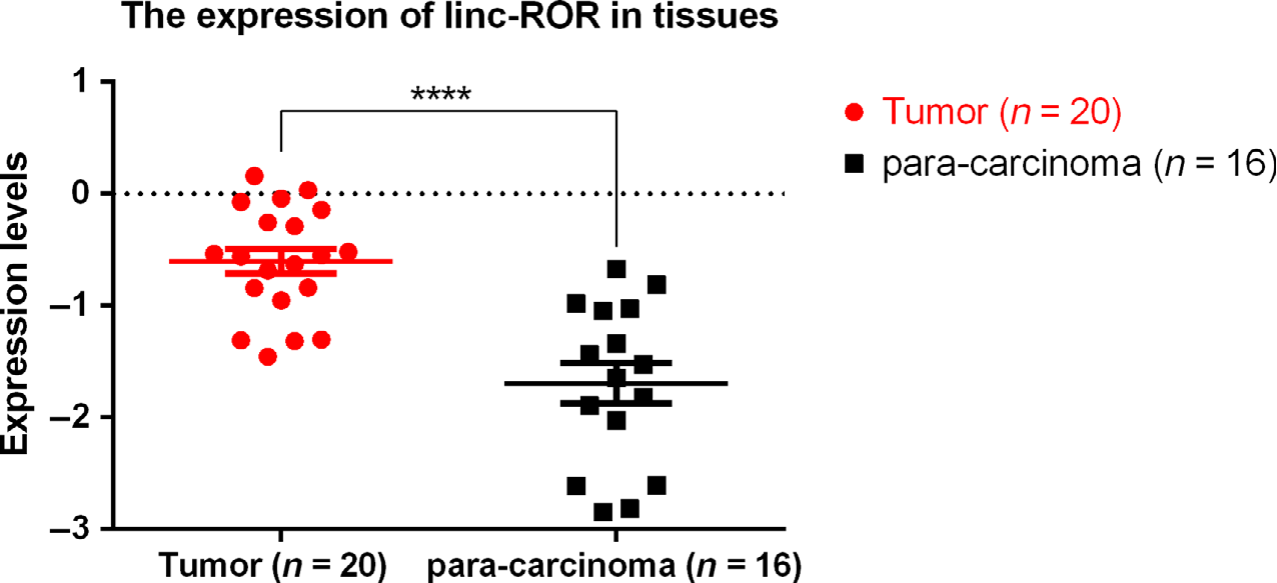
linc‐ROR was upregulated in breast tumor tissues compared with paracarcinoma tissues. Expression level: log( $2^{-\Delta C_{T}}$); ****P < 0.0001; error bars represent SEM.

The RT–qPCR results revealed that the expression of linc‐ROR was significantly upregulated in breast tumor tissues compared with paracarcinoma tissues (*P* < 0.0001) (Fig. 1).

#### 3.1.2 linc‐ROR promotes the proliferation, migration, and invasion of breast cancer cells

**Fig. 2:**
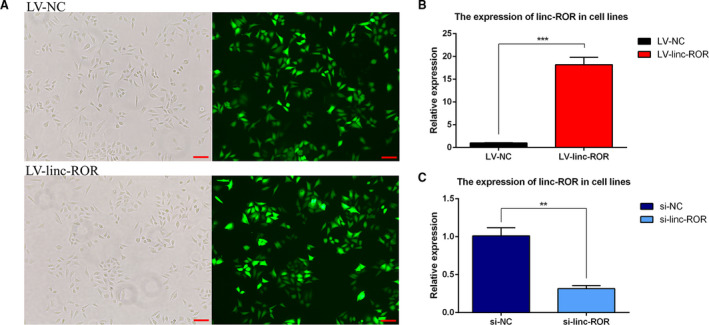
(A, B) The validation of constructed linc‐ROR‐overexpressing (LV‐linc‐ROR) cell lines. (A) The efficiency of lentivirus vector transfection was observed on the same view under the microscope and the fluorescence microscope. Almost all the cells expressed green fluorescence; scale bar = 100 μm. (B) RT–qPCR validation. linc‐ROR was significantly upregulated in LV‐linc‐ROR compared with LV‐NC. (C) RT–qPCR validation of constructed linc‐ROR knockdown (si‐linc‐ROR) cell lines. linc‐ROR was significantly downregulated in si‐linc‐ROR compared with si‐NC. **P < 0.01, ***P < 0.001; error bars represent SEM.

Under the fluorescence microscope, almost all the lentivirus vector‐transfected cells exhibited green fluorescence (Fig. 2A), and RT–qPCR analysis of the RNA extracted from the transfected cells revealed that linc‐ROR was expressed nearly 20 times higher in LV‐linc‐ROR than in LV‐NC (*P* < 0.001) (Fig. 2B). Additionally, after transfection with the lncRNA smart silencer, the expression of linc‐ROR in si‐linc‐ROR decreased by nearly 65% compared with that in si‐NC (*P* < 0.01) (Fig. 2C). The linc‐ROR overexpression and knockdown cell models were successfully constructed.

**Fig. 3:**
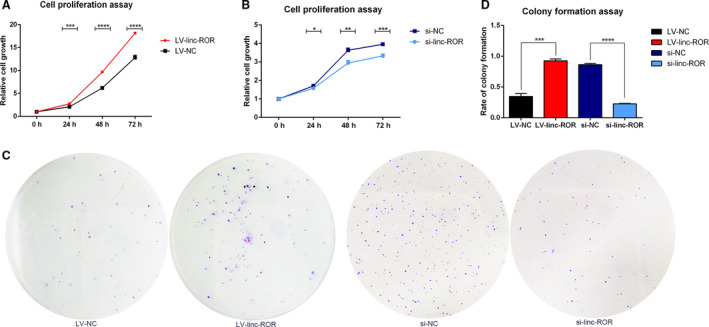
linc‐ROR promoted the proliferation of breast cancer cells. (A, B) linc‐ROR promoted the proliferation of breast cancer cells shown by CCK8 assay. (C, D) linc‐ROR promoted the colony formation of breast cancer cells. *P < 0.05, **P < 0.01, ***P < 0.001, ****P < 0.0001; error bars represent SEM.

Cell proliferation assay was performed to examine the role of linc‐ROR in breast cancer cell proliferation. By detecting the absorbance every 24 h for a total of 3 days and normalizing it to the absorbance at 0 h, LV‐linc‐ROR was shown to grow significantly faster than LV‐NC (Fig. 3A); conversely, si‐NC grew significantly slower than si‐linc‐ROR (Fig. 3B). These results demonstrated that linc‐ROR could promote the proliferation of breast cancer cells. The plate colony formation assay also suggested that LV‐linc‐ROR promoted the colony formation of breast cancer cells and that si‐linc‐ROR suppressed it; these results were obtained either by directly observing the colony after staining (Fig. 3C) or by counting the number of colonies (Fig. 3D).

**Fig. 4:**
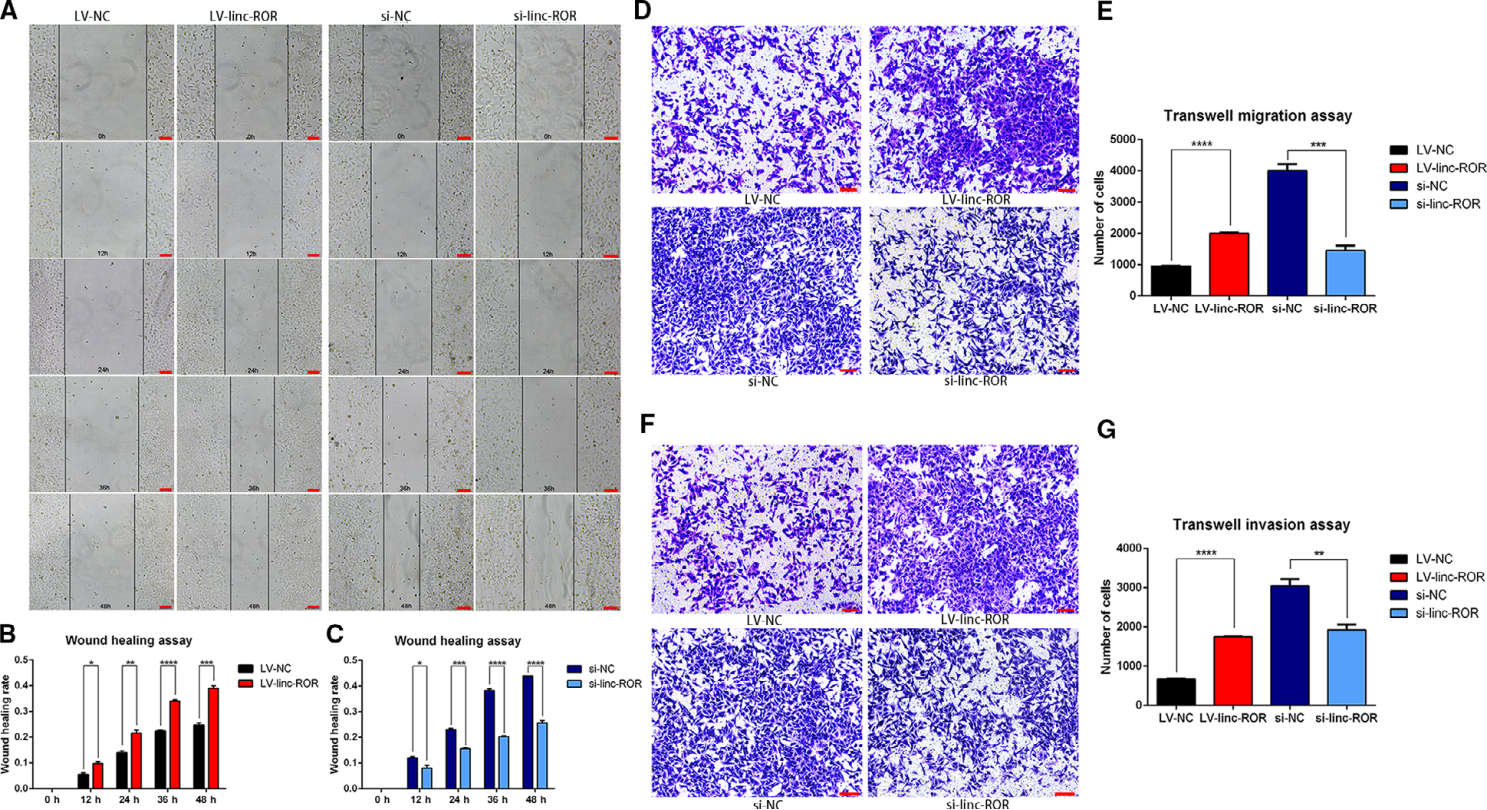
linc‐ROR promoted the migration and invasion of breast cancer cells. (A–C) Wound healing assay revealed that linc‐ROR promoted the migration of breast cancer cells. Pictures were taken at 0, 12, 24, 36, and 48h, respectively, under a microscope at 10× magnification, scale bar = 100 μm, and the wound healing rate was calculated. (D, E) Transwell migration assay revealed that linc‐ROR promoted the migration of breast cancer cells. Pictures of cell migration were taken under the microscope at 10× magnification, scale bar = 100 μm, and the quantitative analysis of the migration of cells was performed. (F, G) Transwell invasion assay revealed that linc‐ROR promoted the invasion of breast cancer cells. Pictures of cell invasion were taken under the microscope at 10× magnification, scale bar = 100 μm, and the quantitative analysis of the invasion of cells was performed. *P < 0.05, **P < 0.01, ***P < 0.001, ****P < 0.0001; error bars represent SEM.

By a wound healing assay, cell migration was observed every 12 h for 2 days, and images were taken under a microscope at 10× magnification (Fig. 4A); LV‐linc‐ROR showed significantly higher wound healing rates than LV‐NC in every 12 h (Fig. 4B), and si‐linc‐ROR indicated significantly lower wound healing rates than si‐NC in every 12 h (Fig. 4C). In addition, a transwell assay was conducted to explore the migration and invasion ability of these cells. The cells were observed after staining and counted under the microscope at 10× magnification, LV‐linc‐ROR had significantly more cells passing through the transwell chamber than LV‐NC, and conversely, si‐linc‐ROR had significantly fewer cells passing through the transwell chamber than si‐NC, in both the migration (Fig. 4D,E) and invasion (Fig. 4F,G) assays. The results from both sides suggested that linc‐ROR could promote cell migration and invasion in breast cancer.

#### 3.1.3 linc‐ROR decreases the sensitivity of breast cancer cells to rapamycin and downregulates the expression of mTOR

**Fig. 5:**
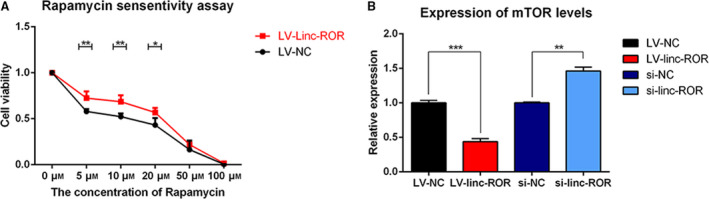
linc‐ROR decreased the sensitivity of breast cancer cells to rapamycin and downregulated the expression of mTOR. (A) Cell viability of LV‐linc‐ROR compared with LV‐NC under rapamycin treatment for 72h with the concentration of 0, 5, 10, 20, 50, and 100 μm, respectively; (B) the expression level of mTOR in LV‐linc‐ROR compared with LV‐NC and si‐linc‐ROR compared with si‐NC. *P < 0.05, **P < 0.01, ***P < 0.001; error bars represent SEM.

When LV‐linc‐ROR and LV‐NC were incubated in the same way with a series of concentrations of rapamycin, LV‐linc‐ROR showed significantly higher cell viability than LV‐NC, especially at concentrations of 5, 10, and 20 μm (Fig. 5A). Since mTOR is the target of rapamycin, the effect of linc‐ROR on the expression of mTOR was determined by RT–qPCR. The result suggested that the overexpression of linc‐ROR downregulated the expression of mTOR, while the knockdown of linc‐ROR upregulated it (Fig. 5B).

### 3.2 Differentially expressed RNAs in LV‐linc‐ROR compared with LV‐NC

**Fig. 6:**
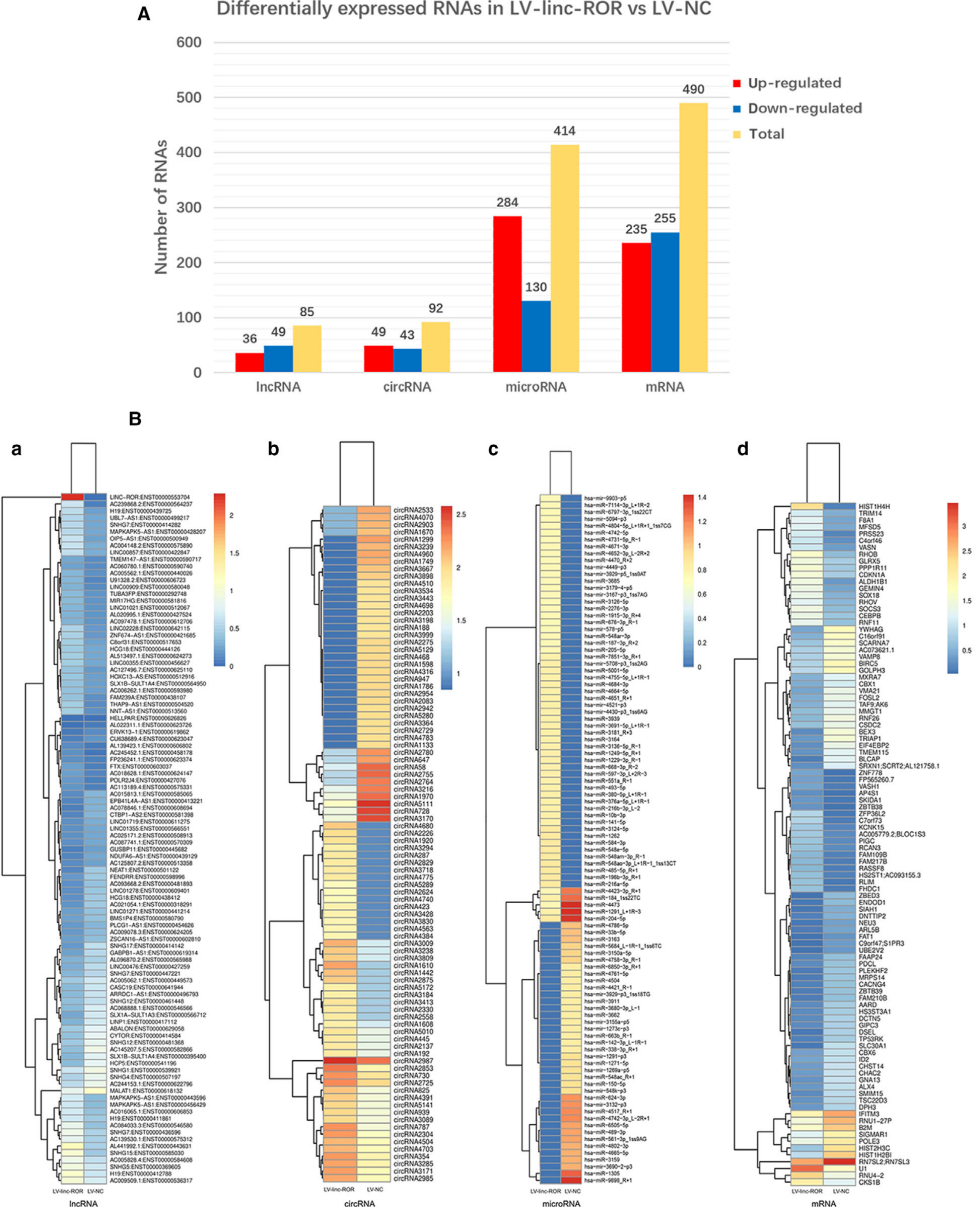
Differentially expressed RNAs in LV‐linc‐ROR compared with LV‐NC. (A) The amount of differentially expressed lncRNAs, circRNAs, microRNAs, and mRNAs in LV‐linc‐ROR compared with LV‐NC. Red indicated upregulated RNAs, blue indicated downregulated RNAs, and yellow indicated the total number of them. (B) Heatmaps of the top 100 most differentially expressed lncRNAs (a), circRNAs (b), microRNAs (c), and mRNAs (d) in LV‐linc‐ROR compared with LV‐NC. Red indicated a high expression level, and blue indicated a low expression level.

To understand the underlying mechanism of how linc‐ROR acts as an oncogene and decreases the sensitivity of breast cancer cells to rapamycin, the whole transcriptome sequencing was conducted on LV‐linc‐ROR and LV‐NC. As shown in Fig. 6, there were 85 lncRNAs, 92 circRNAs, 414 microRNAs, and 490 mRNAs that were differentially expressed in LV‐linc‐ROR compared with LV‐NC. (Fig. 6).

**Fig. 7:**
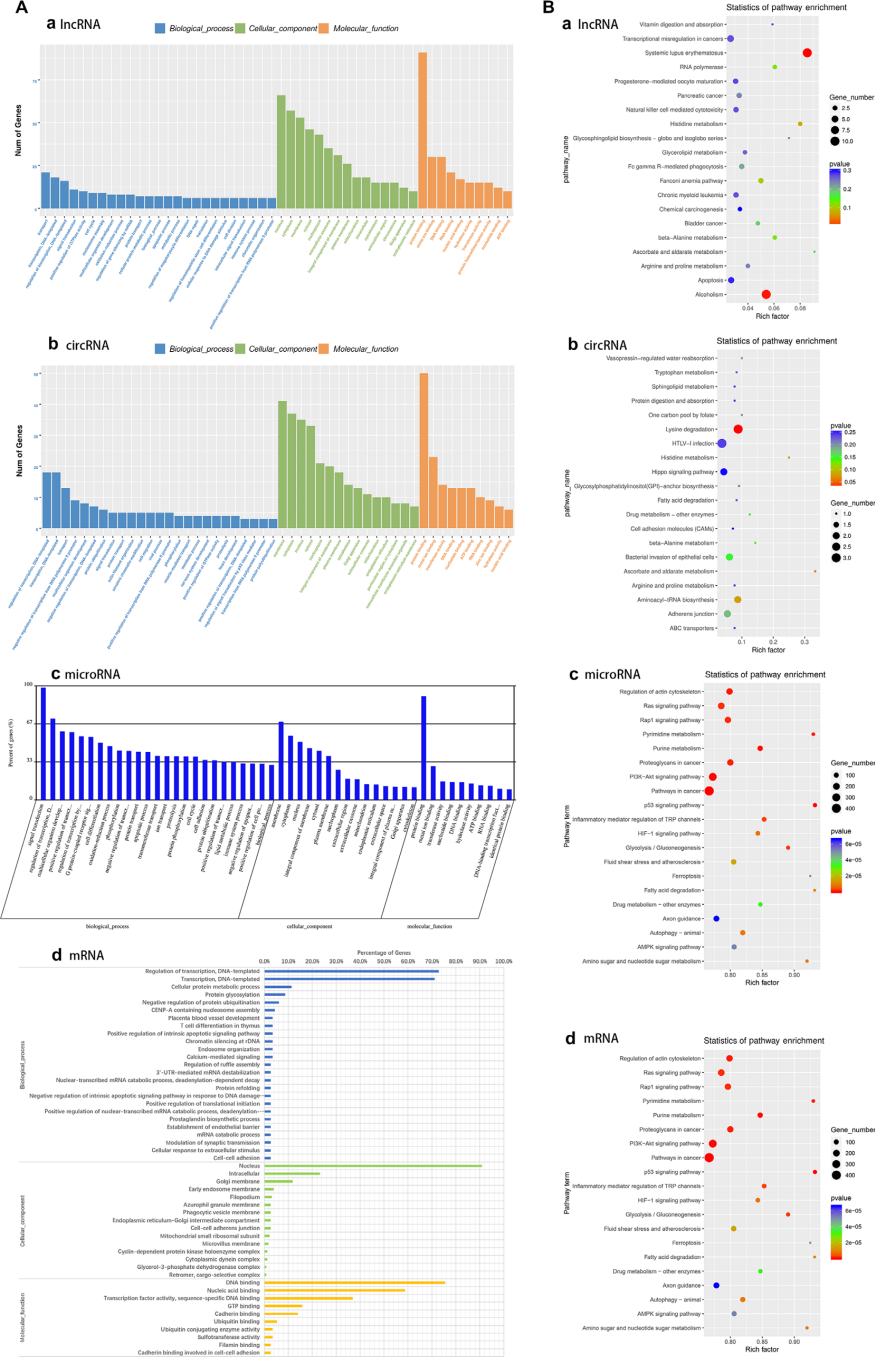
GO analysis (A) and KEGG pathway analysis (B) of target genes of differentially expressed lncRNAs (a), circRNAs (b), microRNAs (c), and mRNAs (d) in LV‐linc‐ROR compared with LV‐NC.

In addition, GO analyses were performed from three aspects: biological process, cellular component, and molecular function. The target genes of all the differentially expressed RNAs (DE‐RNAs) in LV‐linc‐ROR compared with LV‐NC were mainly enriched in the biological processes of ‘transcription, DNA‐templated’, ‘regulation of transcription, DNA‐templated’, ‘protein transport’, ‘signal transduction’, ‘apoptotic process’, ‘cell cycle’, etc. (Fig. 7A). In particular, the target genes of DE‐lncRNAs were also enriched in the biological process of ‘regulation of gene silencing by miRNA’ (Fig. 7A‐a); those of DE‐mRNAs were enriched in the biological processes of ‘3′‐UTR‐mediated mRNA destabilization’, ‘positive regulation of translation initiation’, ‘mRNA catabolic process’ (Fig. 7A‐d). These results indicated the possibility that linc‐ROR may affect the biological process of breast cancer cells through a ceRNA mechanism. Additionally, through KEGG analyses, these target genes were found to be mainly enriched in the pathways of ‘pathways in cancer’, ‘PI3K‐Akt signaling pathway’, ‘drug metabolism’, and ‘autophagy’. (Fig. 7B).

### 3.3 linc‐ROR serves as a ceRNA sponge for miR‐194‐3p and regulates MECP2 in breast cancer

**Fig. 8:**
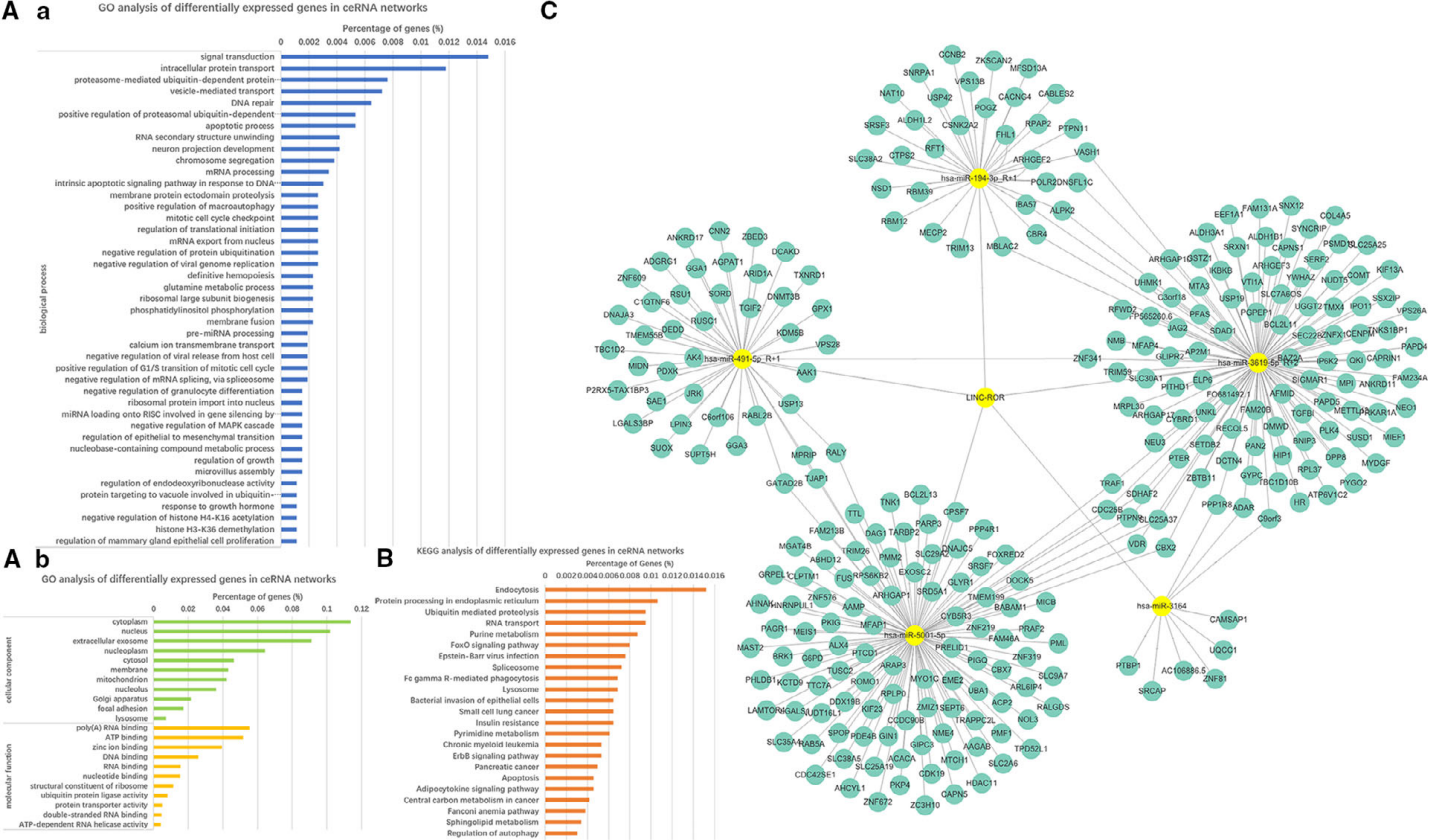
ceRNA network analysis. (A) GO analysis of target genes from ceRNA network analysis was performed, including three aspects: ‘biological process’ (a), ‘cellular component’, and ‘molecular function’ (b). (B) KEGG analysis of target genes from ceRNA network analysis. (C) ceRNA networks which included linc‐ROR with its relative hsa‐miRNAs and mRNAs were drawn by Cytoscape software.

To further explore the possibility of linc‐ROR exhibiting a ceRNA regulatory mechanism, the DE‐RNAs were subjected to ceRNA network analysis. A series of ceRNA combinations were screened out and then chosen to perform GO and KEGG pathway analyses. It was found that these ceRNA combinations were mainly involved in the biological processes of ‘apoptotic process’, ‘regulation of translational initiation’, ‘regulation of EMT’, ‘regulation of growth’, ‘regulation of mammary gland epithelial cell proliferation’, etc. (Fig. 8A), and participated in the pathways of ‘RNA transport’, ‘apoptosis’, ‘regulation of autophagy’, etc. (Fig. 8B). Then, these ceRNA combinations, which included linc‐ROR, were illustrated in a network diagram with their relevant hsa‐microRNAs and mRNAs (Fig. 8C). linc‐ROR could function as the ceRNA sponge for miR‐194‐3p, miR‐3164, miR‐3619‐5p, miR‐491‐5p, and miR‐5001‐5p to regulate their respective target mRNAs. Subsequently, considering the most likely binding site of linc‐ROR and the current research status of the above five microRNAs, miR‐194‐3p was chosen for further study.

**Fig. 9:**
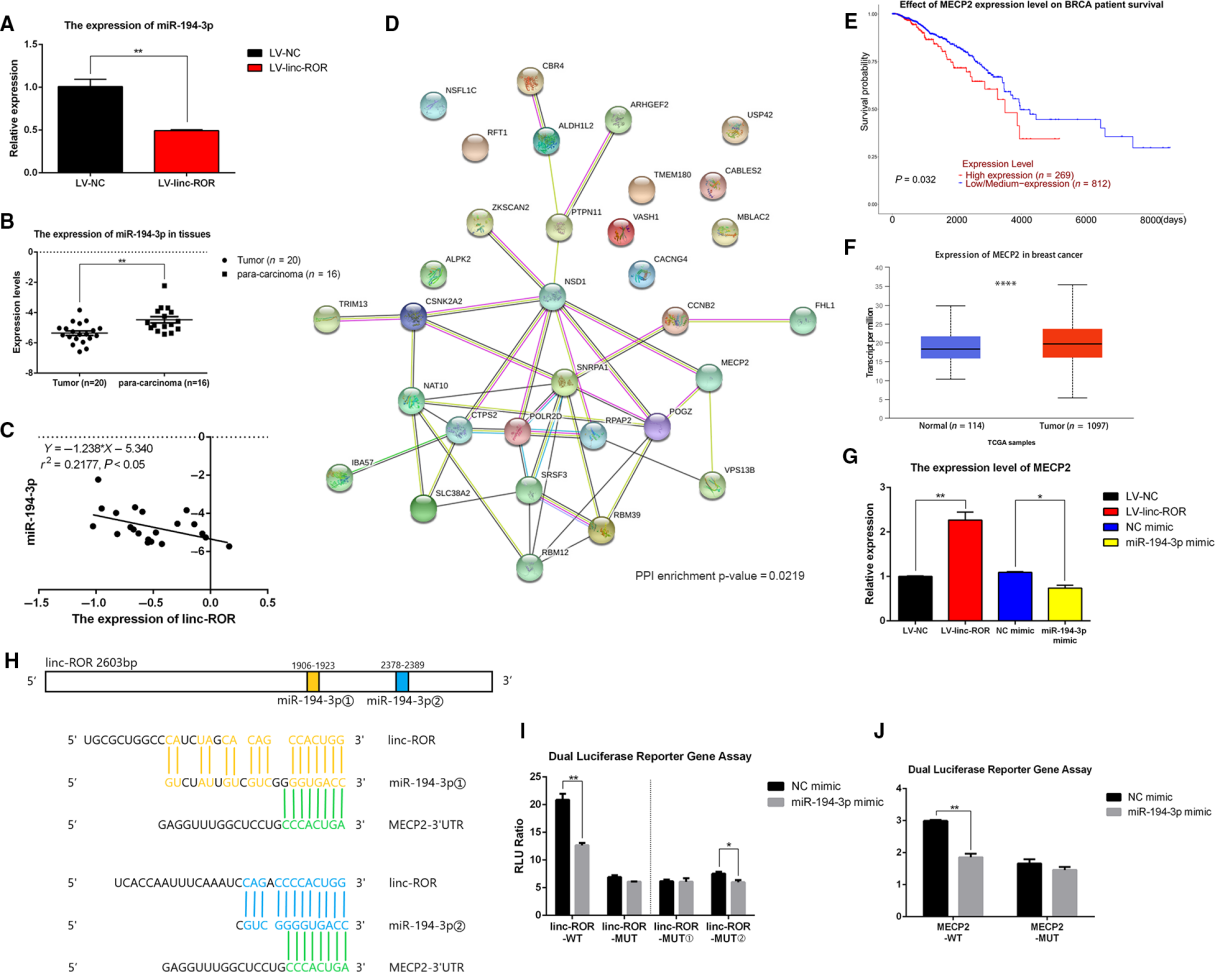
linc‐ROR acted as the sponge of miR‐194‐3p and regulated MECP2. (A–C) The expression level of miR‐194‐3p was negatively regulated by linc‐ROR in breast cancer cells and tissues. (A) miR‐194‐3p was downregulated in linc‐ROR‐overexpressing (LV‐linc‐ROR) cells than LV‐NC; (B) miR‐194‐3p was downregulated in breast cancer tissues than paracarcinoma tissues; (C) the expression level of miR‐194‐3p was negatively correlated with the expression level of linc‐ROR in breast cancer tissues. (D) PPI network analysis of target genes of miR‐194‐3p. (E–G) MECP2 acted as an oncogene in breast cancer, and the expression level of MECP2 was negatively regulated by miR‐194‐3p and positively regulated by linc‐ROR in breast cancer. (E) Effect of MECP2 expression on breast cancer patients survival was analyzed by using UALCAN database, and high expression level of MECP2 decreased the survival rate of breast cancer patients; (F) the expression level of MECP2 in breast tumor tissues was analyzed by using UALCAN database, and MECP2 was significantly upregulated in breast tumor tissues than normal tissues; (G) the expression level of MECP2 was upregulated in linc‐ROR‐overexpressing (LV‐linc‐ROR) cell lines and downregulated in miR‐194‐3p‐overexpressing (miR‐194‐3p mimic) cell lines. (H) Schematic graph of putative binding sites of miR‐194‐3p in the linc‐ROR or the 3′‐UTR region of MECP2; (I) dual‐luciferase reporter gene assay after co‐transfecting cells with linc‐ROR‐WT/MUT and miR‐194‐3p mimic/NC mimic. ‘linc‐ROR‐MUT’: All the sequences of predicted binding sites were mutated, ‘linc‐ROR‐MUT①’: Sequences of binding site ① on linc‐ROR were mutated; ‘linc‐ROR‐MUT②’: Sequences of binding site ② on linc‐ROR were mutated; ‘RLU Ratio’: firefly fluorescence value/Renilla fluorescence value; (J) dual‐luciferase reporter gene assay after co‐transfecting cells with MECP2‐WT/MUT and miR‐194‐3p mimic/NC mimic. *P < 0.05, **P < 0.01, ***P < 0.001, ****P < 0.0001; error bars represent SEM.

According to RT–qPCR validation, miR‐194‐3p was significantly downregulated in the linc‐ROR‐overexpressing cell line (LV‐linc‐ROR) (Fig. 9A) and significantly downregulated in the breast cancer tissues that expressed high levels of linc‐ROR (Fig. 9B). Through correlation analysis, we found that the expression of miR‐194‐3p was negatively correlated with the expression of linc‐ROR in breast cancer tissues (Fig. 9C). That is, linc‐ROR could downregulate the expression of miR‐194‐3p in breast cancer. Taking the target genes of miR‐194‐3p for PPI network analysis, we found that *NSD1, SNRPA1, SRSF3, RBM39, MECP2*, etc., were the main regulatory genes (Fig. 9D). Analysis of data from 1081 breast cancer patients in the UALCAN database showed that those with high expression of MECP2 showed a lower probability of survival (Fig. 9E), and by comparing the expression levels of 1097 breast tumor tissues and 114 normal tissues, MECP2 was found to be upregulated in breast tumor tissues compared with normal tissues (Fig. 9F). These results revealed that MECP2 functioned as an oncogene in breast cancer. In addition, RT–qPCR validated that MECP2 was upregulated in the linc‐ROR‐overexpressing cell line (LV‐linc‐ROR) and downregulated in the miR‐194‐3p‐overexpressing cell line (miR‐194‐3p mimic) (Fig. 9G), which suggested that MECP2 was positively regulated by linc‐ROR and negatively regulated by miR‐194‐3p. Therefore, we inferred that linc‐ROR could function as an onco‐lncRNA by serving as a ceRNA sponge for miR‐194‐3p and then regulating its target MECP2.

To explore the possibility of binding between linc‐ROR and miR‐194‐3p, and between miR‐194‐3p and the 3′‐UTR of MECP2, we performed a dual‐luciferase reporter gene assay. Two binding sites of linc‐ROR and miR‐194‐3p were predicted by DIANA‐LncBase, namely binding site ① (1906–1923 bp on linc‐ROR) and binding site ② (2378–2389 bp on linc‐ROR). Additionally, the binding sites of miR‐194‐3p on the MECP2‐3′‐UTR were predicted by TargetScan. All of the above sites were combined on the sequence of miR‐194‐3p (Fig. 9H). Then, the linc‐ROR‐WT/MUT and MECP2‐WT/MUT plasmids were constructed and used to perform dual‐luciferase reporter gene assays. HEK‐293T cells cotransfected with linc‐ROR‐WT/MUT, and the miR‐194‐3p mimic or the NC mimic was cultured for 48 h, and then, RLU ratio was determined. The overexpression of miR‐194‐3p decreased the RLU ratio of linc‐ROR‐WT but had no effect on that of linc‐ROR‐MUT (Fig. 9I). These results proved that linc‐ROR could bind to miR‐194‐3p. However, it should also be mentioned that only one binding site of linc‐ROR and miR‐194‐3p was proven to be correct, although two were predicted. Although the base sequence from 2378 to 2389 bp on linc‐ROR (predicted binding site ②) was mutated, the overexpression of miR‐194‐3p could still decrease the RLU ratio of linc‐ROR‐MUT ②, similar to linc‐ROR‐WT, but it had no effect on the RLU ratio of linc‐ROR‐MUT ①, whose sequence from 1906 to 1923 bp (predicted binding site ①) was mutated, and linc‐ROR‐MUT (both binding sites were mutated). These results suggested that the sequence from 1906 to 1923 bp was the real binding site of linc‐ROR and miR‐194‐3p. Likewise, MECP2‐WT/MUT and the miR‐194‐3p mimic or the NC mimic were cotransfected into HEK‐293T cells, and the results of the dual‐luciferase reporter assay showed that the overexpression of miR‐194‐3p decreased the RLU ratio of MECP2‐WT but not that of MECP2‐MUT (Fig. 9J). This result proved that miR‐194‐3p could bind to MECP2‐3′‐UTR. Therefore, we verified that linc‐ROR could function as a sponge for miR‐194‐3p and prevent the inhibitory effect of miR‐194‐3p on MECP2.

### 3.4 linc‐ROR promotes breast cancer progression and decreases the sensitivity to rapamycin through the linc‐ROR/miR‐194‐3p/MECP2 regulatory axis

**Fig. 10:**
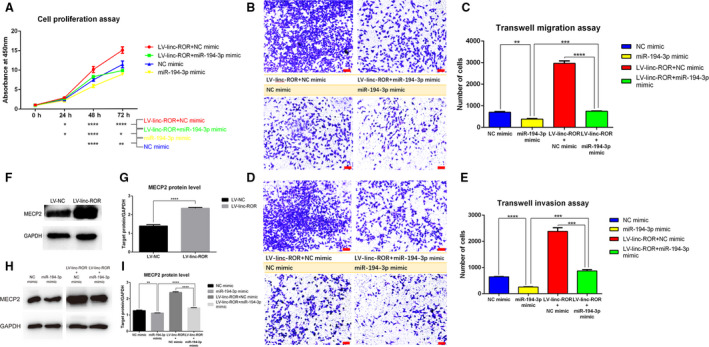
linc‐ROR promoted cell proliferation, migration, and invasion of breast cancer through linc‐ROR/miR‐194‐3p/MECP2 regulatory axis. (A) CCK8 proliferation assay revealed that the overexpression of miR‐194‐3p inhibited cell growth and the rescue of miR‐194‐3p reduced the promoting effect of linc‐ROR on cell growth. (B–C) Transwell migration assay indicated that the overexpression of miR‐194‐3p inhibited the cell migration and the rescue of miR‐194‐3p expression reduced the promoting effect of linc‐ROR on it. Pictures were captured under the microscope at 10× magnification, scale bar = 100 μm, and the quantitative analysis of the migration assay was performed. (D, E) Transwell invasion assay indicated that the overexpression of miR‐194‐3p inhibited cell invasion and the rescue of miR‐194‐3p expression reduced the promoting effect of linc‐ROR on it. Pictures were captured under the microscope at 10× magnification, scale bar = 100 μm, and the quantitative analysis of the invasion assay was performed. (F–I) Western blot assays determined the expression level of MECP2 protein in cell lines, pictures of protein bands were captured, and the gray value of them was calculated by Image J. (F, G) linc‐ROR upregulated the protein level of MECP2; (H, I) miR‐194‐3p downregulated the protein level of MECP2, and the rescue of miR‐194‐3p reduced the upregulating effect of linc‐ROR on MECP2. ‘miR‐194‐3p mimic/NC mimic’: overexpression of miR‐194‐3p/its negative control in MCF‐7 cell line, ‘LV‐linc‐ROR + miR‐194‐3p mimic/NC mimic’: rescue/not rescue the expression of miR‐194‐3p in linc‐ROR‐overexpressing MCF‐7 cell line; *P < 0.05, **P < 0.01, ***P < 0.001, ****P < 0.0001; error bars represent SEM.

To study the effect of the linc‐ROR/miR‐194‐3p/MECP2 regulatory axis on breast cancer cells, we transfected LV‐linc‐ROR (overexpression of linc‐ROR in MCF‐7) or MCF‐7 cells with the miR‐194‐3p mimic or the NC mimic, respectively. Four groups of cell lines were constructed, namely ‘miR‐194‐3p mimic’ (overexpression of miR‐194‐3p), ‘NC mimic’ (negative control), ‘LV‐linc‐ROR + miR‐194‐3p mimic’ (rescued the low expression of miR‐194‐3p in LV‐linc‐ROR), and ‘LV‐linc‐ROR + NC mimic’ (not rescued the low expression of miR‐194‐3p in LV‐linc‐ROR). First, we determined the proliferation status of these cell lines and found that the overexpression of miR‐194‐3p inhibited the proliferation of the breast cancer cells and reduced the promoting effect of linc‐ROR on proliferation (Fig. 10A). Then, transwell assays indicated that the overexpression of miR‐194‐3p inhibited the migration (Fig. 10B,C) and invasion (Fig. 10D,E) abilities of breast cancer cells, and reduced the effect of linc‐ROR on promoting these abilities. Since linc‐ROR could downregulate the expression of miR‐194‐3p which functions as a tumor suppressor miRNA, and the rescue of miR‐194‐3p expression in LV‐linc‐ROR reduced the promoting effect of linc‐ROR, we concluded that linc‐ROR promoted the proliferation, migration, and invasion of breast cancer cells by functioning as a ceRNA sponge for miR‐194‐3p.

In our previous study, MECP2 acted as an oncogene in breast cancer, and its expression was positively regulated by linc‐ROR but negatively regulated by miR‐194‐3p. We also detected MECP2 protein levels in breast cancer cells that transfected with linc‐ROR and/or the miR‐194‐3p mimic/NC mimic. The results of the western blot assay suggested that linc‐ROR could upregulate the MECP2 protein level (Fig. 10F,G). In addition, the overexpression of miR‐194‐3p downregulated the protein level of MECP2 and reduced the linc‐ROR‐mediated upregulation of the MECP2 protein (Fig. 10H,I). This result showed the possibility that linc‐ROR functioned as an onco‐lncRNA by reducing the silencing effect of miR‐194‐3p on MECP2.

**Fig. 11:**
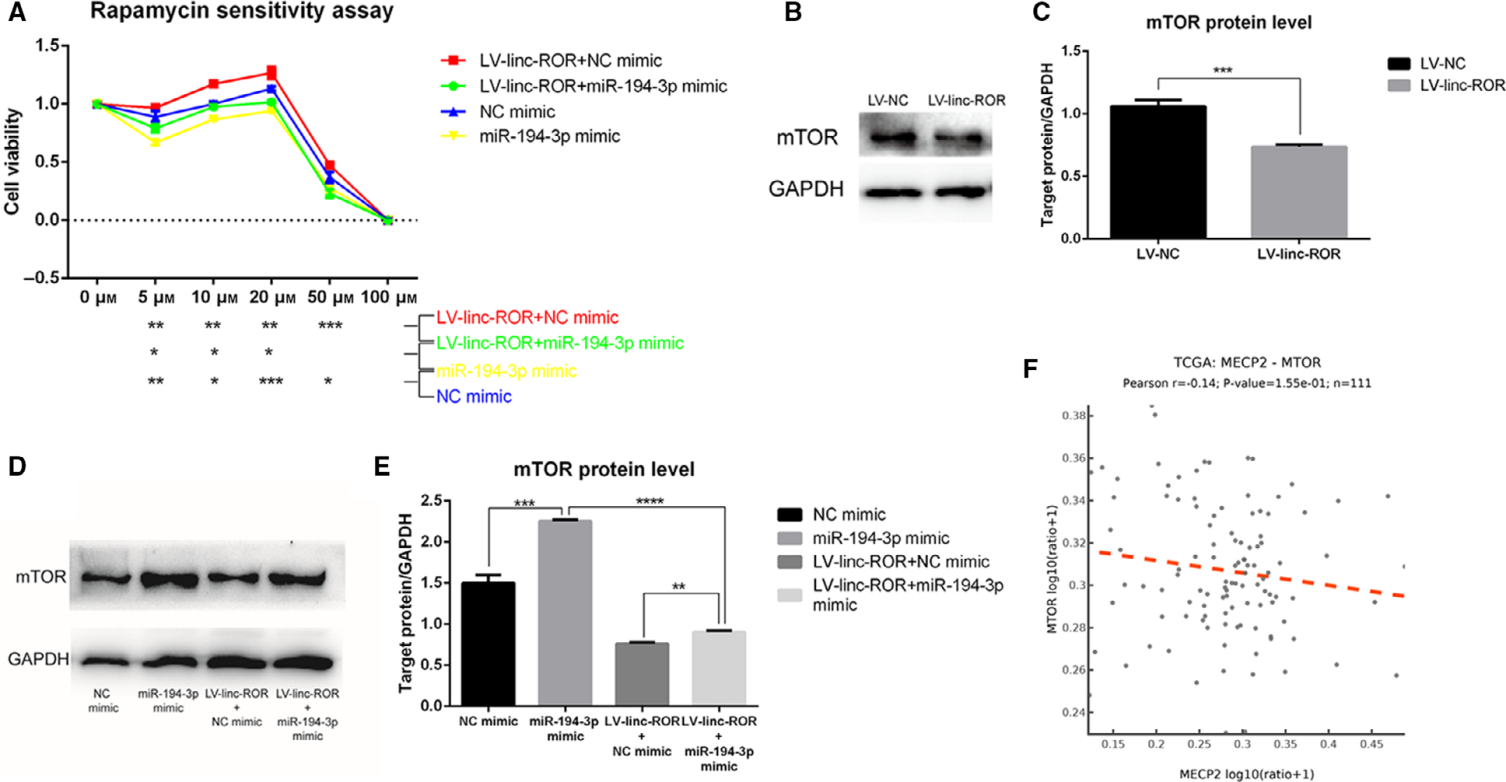
linc‐ROR decreased the sensitivity of breast cancer cells to rapamycin through linc‐ROR/miR‐194‐3p/MECP2 regulatory axis. (A) CCK8 cell viability assay revealed that the overexpression of miR‐194‐3p group had lower cell viability than negative control and the rescue of miR‐194‐3p expression reduced the inhibiting effect of linc‐ROR on it. (B–E) Western blot assays determined the expression level of mTOR protein, pictures of protein bands were captured, and the gray value of them was calculated by Image J. (B, C) linc‐ROR downregulated the protein level of mTOR; (D, E) miR‐194‐3p upregulated the protein level of mTOR, and the rescue of miR‐194‐3p expression reduced the downregulating effect of linc‐ROR on it. (F) The correlation analysis of the expression level of MECP2 and mTOR from TCGA database. ‘miR‐194‐3p mimic/NC mimic’: overexpression of miR‐194‐3p/its negative control in MCF‐7 cell line, ‘LV‐linc‐ROR + miR‐194‐3p mimic/NC mimic’: rescue/not rescue the expression of miR‐194‐3p in linc‐ROR‐overexpressing MCF‐7 cell line; *P < 0.05, **P < 0.01, ***P < 0.001, ****P < 0.0001; error bars represent SEM.

Since the overexpression of linc‐ROR was found to decrease the sensitivity of breast cancer cells to rapamycin, the miR‐194‐3p mimic or NC mimic was transfected into MCF‐7 cells and LV‐linc‐ROR cells to explore the underlying regulatory mechanism. Four groups of cell lines were cultured with a series of concentrations of rapamycin. The overexpression of miR‐194‐3p increased the sensitivity of breast cancer cells to rapamycin, thus reducing cell viability in the presence of rapamycin, and the rescued miR‐194‐3p expression reduced the linc‐ROR‐mediated decrease in the sensitivity of breast cancer cells to rapamycin (Fig. 11A). Since mTOR is the target of rapamycin, its protein level in these cell lines was also detected through western blot assay. linc‐ROR could downregulate the protein level of mTOR (Fig. 11B,C), and the overexpression of miR‐194‐3p upregulated the protein level of mTOR and reduced the inhibitory effect of linc‐ROR on the expression of the mTOR protein (Fig. 11D,E). This result suggested that linc‐ROR could downregulate mTOR protein levels by silencing the positive effect of miR‐194‐3p on mTOR, thus reducing the target of rapamycin and promoting breast cancer cell survival in the presence of rapamycin. In addition, 111 cases retrieved from TCGA using the AIPuFu platform (www.aipufu.com) showed that the expression level of mTOR in breast cancer tissues was negatively correlated with the expression of MECP2 (Fig. 11F). We can speculate that linc‐ROR promotes breast cancer cells to survive in rapamycin treatment by functioning as a ceRNA sponge for miR‐194‐3p, removing its inhibitory effect on MECP2 and downregulating the expression level of mTOR.

## 4 Discussion

### 4.1 linc‐ROR promotes cell proliferation, migration, and invasion through the linc‐ROR/miR‐194‐3p/MECP2 regulatory axis in breast cancer

Increased evidence has proven the important role of lncRNAs in breast cancer. In our study, linc‐ROR was found to be upregulated in breast cancer tissues compared with paracarcinoma tissues, indicating its carcinogenic potential. By upregulating and downregulating the expression level of linc‐ROR in breast cancer cell lines, we found that the overexpression of linc‐ROR could promote cell proliferation, colony formation, and cell migration, and invasion, while reducing expression had the opposite effects.

Through whole transcriptome sequencing, 85 lncRNAs, 414 microRNAs, 490 mRNAs, and 92 circRNAs were found to be differentially expressed in the linc‐ROR overexpression group versus the NC group. The target genes of these DE‐RNAs were subjected to GO and KEGG pathway analyses, and the results revealed the possibility that linc‐ROR may affect the development and progression of breast cancer through a ceRNA mechanism. Then, we subjected the differentially expressed lncRNAs, hsa‐miRNAs, circRNAs, and mRNAs to ceRNA mechanism analysis and found that these ceRNA networks were mainly enriched in the biological processes of ‘apoptotic process’, ‘regulation of EMT’, ‘regulation of growth’, and ‘regulation of mammary gland epithelial cell proliferation’, indicating the regulatory effect of these ceRNA networks in breast cancer cells.

In addition, we found that linc‐ROR could function as a ceRNA sponge for miR‐194‐3p, miR‐3164, miR‐3619‐5p, miR‐491‐5p, and miR‐5001‐5p. Among these five miRNAs, miR‐3164 was found to participate in the ceRNA mechanism of circRAD18 to promote tumor progression in triple‐negative breast cancer (Zou *et al*., 2019); miR‐3619‐5p was reported to be involved in the ceRNA regulatory networks of linc01410 in papillary thyroid carcinoma (Wang *et al*., 2019), linc00342 in infantile hemangioma (Liu *et al*., 2019), linc00202 in retinoblastoma (Yan *et al*., 2019), and circZFR in hepatocellular carcinoma (Tan *et al*., 2019); miR‐491‐5p was found to be an inhibitor both in ERα‐positive breast cancer (Hui *et al*., 2015) and in HER2‐positive breast cancer cells (Leivonen *et al*., 2014). There were limited studies about miR‐5001‐5p in breast cancer.

For miR‐194‐3p, it is reported that SLC12A5 could function as a ceRNA sponge for miR‐194‐3p to regulate TWIT1 expression, thus exerting oncogenic function in lung adenocarcinoma (Xia *et al*., 2019); lncRNA PTPRG‐AS1 could act as a miR‐194‐3p sponge to regulate the metastasis of nasopharyngeal carcinoma cells (Yi *et al*., 2019); and CG200745 could inhibit cholangiocarcinoma growth by upregulating the expression of miR‐194‐3p (Jung *et al*., 2017). Also, miR‐194 could function as a tumor suppressor in a triple‐negative breast cancer (Javadian *et al*., 2019) and HER2‐overexpressing human breast cancer (Le *et al*., 2012). But the studies about the ceRNA regulatory mechanism of miR‐194‐3p in breast cancer are rare. According to RT–qPCR result, we found that miR‐194‐3p was significantly downregulated in breast cancer tissues and the expression level of miR‐194‐3p was negatively correlated with linc‐ROR. These results preliminarily indicated that linc‐ROR could function as a ceRNA sponge for miR‐194‐3p. Then, through PPI interaction analysis of the target mRNAs of miR‐194‐3p, MECP2 (methyl‐CpG‐binding protein 2) was screened out for further study.

MECP2 is reported to be an oncogene in breast cancer. Three polymorphisms in strong linkage disequilibrium located in MECP2 gene regions were related to breast cancer susceptibility in populations (Sapkota *et al*., 2012). MECP2 mRNA was significantly upregulated in neoplastic tissues compared with non‐neoplastic tissues (Muller *et al*., 2003). MECP2 mediates the hypermethylation of specific CpGs to silence the tumor suppressor KLK6, thus promoting EMT in metastatic breast cancer (Pampalakis *et al*., 2009). In addition, both ERα and ERβ in breast cancer cells are downregulated by promoter methylation and subsequent binding of MECP2 (Wilson and Westberry, 2009). And in ER‐negative MDA‐MB‐231 cells, which are more malignant, displayed higher expression of MECP2, suggesting the association between prominent epigenetic alterations and increased malignant properties of breast cancer cells (Tryndyak *et al*., 2006).

In our study, MECP2 was indicated to decrease the survival rates in breast cancer patients, and MECP2 was significantly upregulated in breast cancer tissues than normal tissues, which was consistent with previous reports. By RT–qPCR validation, the expression of MECP2 was found to be positively regulated by linc‐ROR and negatively regulated by miR‐194‐3p. Through a dual‐luciferase reporter gene assay, we identified that linc‐ROR could function as a ceRNA sponge for miR‐194‐3p by binding to it, and miR‐194‐3p could bind to the 3′‐UTR region of its target gene MECP2. These results suggested that linc‐ROR could function as a sponge for miR‐194‐3p and prevent the inhibitory effect of miR‐194‐3p on MECP2. Then, we confirmed that the overexpression of miR‐194‐3p could inhibit the proliferation, migration, and invasion of breast cancer cells and downregulate the protein level of MECP2, and the restoration of miR‐194‐3p expression could reduce the linc‐ROR‐mediated promotion of breast cancer progression. Therefore, we concluded that linc‐ROR could function as a ceRNA sponge for miR‐194‐3p to downregulate its expression, and then decrease the inhibition of MECP2 by miR‐194‐3p, thus promoting the proliferation, migration, and invasion of breast cancer cells.

### 4.2 linc‐ROR decreases the sensitivity of breast cancer cells to rapamycin through the linc‐ROR/miR‐194‐3p/MECP2 regulatory axis

linc‐ROR, miR‐194, and MECP2 have been reported to affect chemosensitivity or chemoresistance in cancers. For example, linc‐ROR could promote chemotherapy tolerance of breast cancer (Chen *et al*., 2016a), regulate gemcitabine resistance in breast cancer (Chen *et al*., 2016b), regulate chemoresistance in docetaxel‐resistant lung adenocarcinoma cells (Pan *et al*., 2017), and contribute to the resistance of pancreatic cancer cells to gemcitabine (Li *et al*., 2016). miR‐194 influences the sensitivity to docetaxel in cancer therapy (Geretto *et al*., 2017) and increases the chemosensitivity of nonsmall cell lung cancer cells (Zhu *et al*., 2016), and low expression of miR‐194 was observed in chemoresistant hepatocellular carcinoma cells (Ran *et al*., 2019). MECP2 is overexpressed in MCF‐7 cells (Lin and Nelson, 2003) and can be decreased by miR‐1291 to suppress cancer cell proliferation and enhance chemosensitivity (Li *et al*., 2015). The knockdown of MECP2 increased the sensitivity of prostate cancer cells to docetaxel by significantly upregulating GADD45α (Ramachandran *et al*., 2009). A high level of MECP2 was found in two camptothecin (CPT)‐resistant cell lines (CPT30 and KB100) (Ma *et al*., 2008).

Dysregulation of the spatiotemporally controlled mTOR‐driven pathway contributes to breast cancer development, progression, and drug resistance (Butt *et al*., 2019; Guerrero‐Zotano *et al*., 2016). Targeting PI3K/AKT/mTOR‐mediated autophagy is important for increasing the chemosensitivity of tumor cells and preventing drug resistance (Xu *et al*., 2020). Rapamycin is an inhibitor of mTOR that is used as an immunosuppressant in breast cancer therapy. It is necessary to understand the effect of linc‐ROR on drug sensitivity and the mTOR expression by using rapamycin as an indicator. In our study, under different concentrations of rapamycin, the cells overexpressing linc‐ROR showed higher viability than the negative control, and the overexpression of linc‐ROR could downregulate both the mRNA and protein expression levels of mTOR, indicating that linc‐ROR could promote breast cancer cell survival during rapamycin treatment by downregulating the expression of mTOR. It has been reported that the overexpression of mTOR through promoting the transcriptional level of H1F1α leads to increased angiogenesis, which is sensitive to rapamycin treatment (Kasaian *et al*., 2015; Land and Tee, 2007). linc‐ROR likely downregulates the expression of mTOR to decrease angiogenesis and then decrease the sensitivity of cells to rapamycin.

In addition, we found that the overexpression of miR‐194‐3p increased the sensitivity of cells to rapamycin and upregulated the protein level of mTOR, and the restoration of miR‐194‐3p expression reduced the inhibitory effect of linc‐ROR on mTOR protein levels. Additionally, the expression of mTOR was negatively correlated with the expression of MECP2 (*r* = −0.14). This result suggested that linc‐ROR could downregulate the expression of mTOR by functioning as a ceRNA sponge for miR‐194‐3p and preventing its inhibitory effect on MECP2. Similar to the connection between linc‐ROR and angiogenesis, it has also been reported that miR‐194 is a microRNA downstream of p53 that affects the expression of genes that regulates angiogenesis (Brumm *et al*., 2017), miR‐194 is enriched by GO terms including angiogenesis in central nervous system lymphoma (Takashima *et al*., 2019) and could promote angiogenesis in hepatopulmonary syndrome (Chen *et al*., 2019) and in colon cancers (Sundaram *et al*., 2011). MECP2 can reduce the angiogenesis of senescent endothelial progenitor cells (EPCs) through SIRT1 promoter hypermethylation (Wang *et al*., 2018), and SIRT1 promotes angiogenesis by increasing VEGF expression (Balaiya *et al*., 2012). From the above, we can speculate that MECP2 can decrease the expression of mTOR to reduce angiogenesis, but miR‐194 downregulates the expression of MECP2 to promote angiogenesis, thus increasing the sensitivity of breast cancer cells to rapamycin treatment. Since linc‐ROR could function as a ceRNA sponge for miR‐194‐3p and downregulate its expression, we speculate that, through the linc‐ROR/miR‐194‐3p/MECP2 regulatory axis, linc‐ROR could downregulate mTOR levels to decrease angiogenesis and then decrease the sensitivity of breast cancer cells to rapamycin.

## 5 Conclusion

In conclusion, linc‐ROR serves as an onco‐lncRNA in breast cancer and promotes breast cancer cell survival during rapamycin treatment by functioning as a ceRNA sponge for miR‐194‐3p, which targets MECP2.

## Conflict of interest

The authors declare no conflict of interest.

## Author contributions

QZ and XL conceived the project. QZ designed and performed experiments, analyzed the data, and wrote the paper. JG participated in the early period of study. JG, WH, XY, CX, and XL helped to revise the paper. XL supervised the project. All authors read and approved the final manuscript.
